# Long-term effects of lumacaftor/ivacaftor on paranasal sinus abnormalities in children with cystic fibrosis detected with magnetic resonance imaging

**DOI:** 10.3389/fphar.2023.1161891

**Published:** 2023-04-10

**Authors:** Lena Wucherpfennig, Felix Wuennemann, Monika Eichinger, Angelika Seitz, Ingo Baumann, Mirjam Stahl, Simon Y. Graeber, Shengkai Zhao, Jaehi Chung, Jens-Peter Schenk, Abdulsattar Alrajab, Hans-Ulrich Kauczor, Marcus A. Mall, Olaf Sommerburg, Mark O. Wielpütz

**Affiliations:** ^1^ Department of Diagnostic and Interventional Radiology, University Hospital Heidelberg, Heidelberg, Germany; ^2^ Translational Lung Research Center Heidelberg (TLRC), German Center for Lung Research (DZL), Heidelberg, Germany; ^3^ Department of Diagnostic and Interventional Radiology with Nuclear Medicine, University Hospital Heidelberg, Heidelberg, Germany; ^4^ Department of Diagnostic and Interventional Radiology and Neuroradiology, Helios Dr. Horst-Schmidt-Kliniken Wiesbaden, Wiesbaden, Germany; ^5^ Department of Neuroradiology, University Hospital Heidelberg, Heidelberg, Germany; ^6^ Department of Otorhinolaryngology, Head and Neck Surgery, University Hospital Heidelberg, Heidelberg, Germany; ^7^ Department of Pediatric Respiratory Medicine, Immunology and Intensive Care Medicine, Charité-Universitätsmedizin Berlin, Berlin, Germany; ^8^ German Center for Lung Research (DZL), Berlin, Germany; ^9^ Berlin Institute of Health (BIH) at Charité—Universitätsmedizin Berlin, Berlin, Germany; ^10^ Division of Pediatric Pulmonology, Allergy, and Cystic Fibrosis Center, Department of Pediatrics III, University Hospital Heidelberg, Heidelberg, Germany

**Keywords:** cystic fibrosis, magnetic resonance imaging, airway disease, chronic rhinosinusitis, Mucus obstruction

## Abstract

**Introduction:** Chronic rhinosinusitis (CRS) usually presents with nasal congestion, rhinorrhea and anosmia impacts quality of life in cystic fibrosis (CF). Especially mucopyoceles pathognomonic for CRS in CF may cause complications such as spread of infection. Previous studies using magnetic resonance imaging (MRI) demonstrated early onset and progression of CRS from infancy to school age in patients with CF, and mid-term improvements of CRS in preschool and school-age children with CF treated with lumacaftor/ivacaftor for at least 2 months. However, long-term data on treatment effects on paranasal sinus abnomalities in preschool and school-age children with CF are lacking.

**Methods:** 39 children with CF homozygous for F508del (mean age at baseline MRI 5.9 ± 3.0 years, range 1-12 years) underwent MRI before (MRI1) and about 7 months after starting lumacaftor/ivacaftor and then annually (median 3 follow-up MRI, range 1–4) (MRI2-4). MRI were evaluated using the previously evaluated CRS-MRI score with excellent inter-reader agreement. For intraindividual analysis ANOVA mixed-effects analysis including Geisser-Greenhouse correction and Fisher’s exact test, and for interindividual group analysis Mann-Whitney test were used.

**Results:** The CRS-MRI sum score at baseline was similar in children starting lumacaftor/ivacaftor in school age and children starting therapy at preschool age (34.6 ± 5.2 vs.32.9 ± 7.8, *p* = 0.847). Mucopyoceles were the dominant abnormality in both, especially in maxillary sinus (65% and 55%, respectively). In children starting therapy in school age the CRS-MRI sum score decreased longitudinally from MRI1 to MRI2 (−2.1 ± 3.5, *p* < 0.05), MRI3 (−3.0 ± 3.7, *p* < 0.01) and MRI4 (−3.6 ± 4.7, *p* < 0.01), mainly due to a decrease in the mucopyoceles subscore (−1.0 ± 1.5, *p* = 0.059; −1.2 ± 2.0, *p* < 0.05; −1.6 ± 1.8, *p* < 0.01; and −2.6 ± 2.8, *p* = 0.417, respectively). In children starting lumacaftor/ivacaftor in preschool age, the CRS-MRI sum score remained stable under therapy over all three follow-up MRI (0.6 ± 3.3, *p* = 0.520; 2.4 ± 7.6, *p* = 0.994; 2.1 ± 10.5, *p* > 0.999 and −0.5 ± 0.5, *p* = 0.740; respectively).

**Conclusion:** Longitudinal paranasal sinus MRI shows improvements in paranasal sinus abnormalities in children with CF starting lumacaftor/ivacaftor therapy at school age. Further, MRI detects a prevention of an increase in paranasal sinus abnormalities in children with CF starting lumacaftor/ivacaftor therapy at preschool age. Our data support the role of MRI for comprehensive non-invasive therapy and disease monitoring of paranasal sinus abnormalities in children with CF.

## Introduction

Chronic rhinosinusitis (CRS) in patients with cystic fibrosis (CF) is often underrecognized due to the early predominance of pulmonary symptoms, but also significantly contributes to morbidity in CF (Mainz et al., 2009; Berkhout et al., 2013). Moreover, CRS may serve as a reservoir for bacteria and leading to recurrent infections of the lower airways (Eggesbo et al., 2001; Mainz et al., 2009; Berkhout et al., 2013). Previous magnetic resonance imaging (MRI) studies employing a dedicated CRS-MRI scoring system demonstrated a high prevalence of paranasal sinus abnormalities in children with CF from infancy, and an age-dependent increase in prevalence and severity of CRS from infancy to school age (Sommerburg et al., 2020; Wucherpfennig et al., 2022a). The CRS-MRI score correlates with lung disease severity as detected by the chest MRI score in adults (Wucherpfennig et al., 2022b). Further, inflammatory markers from nasal lavage correlate with the CRS-MRI score severity (Chung et al., 2021). These results suggest a need for thorough examination of the paranasal sinuses in children with CF. The recent development of cystic fibrosis transmembrane conductance regulator modulators (CFTRm) significantly improved the therapy of CF abnormalities compared to symptomatic therapies by addressing the disease’s basic defect (De Boeck and Amaral, 2016; Mall et al., 2020). Thus, sinonasal symptoms measured by 22 item Sinonasal Outcome Test (SNOT-22) improved in patients with CF older than 12 years during the first year of elexacaftor/tezacaftor/ivacaftor therapy (Stapleton et al., 2022; Bode et al., 2023). Moreover, in patients with CF 6 years and older inflammatory markers in nasal lavage decreased in the first 12 weeks after therapy initiation with ivacaftor (Mainz et al., 2021). Lumacaftor/ivacaftor was approved in the United States of America 2016 and in the EU in 2018 for the treatment of children with CF homozygous for the F508del mutation aged 6 years and older, and 2018 in the United States of America and 2019 in the EU for children with CF homozygous for the F508del mutation between two and 6 years of age. It was shown that treatment with lumacaftor/ivacaftor results in improvements in clinical parameters such as sweat chloride concentration, thriving, exocrine pancreatic function and lung function, as well as on lung abnormalities detected by chest MRI (Graeber et al., 2021; Hoppe et al., 2021). Moreover, a previous cross-sectional study showed a decrease in the CRS-MRI sum score in children (mean age 9.2 ± 4.4 years, range 3–17 years) with CF homozygous for the F508del mutation after therapy initiation with lumacaftor/ivacaftor at a mid-term follow up after at least 2 months of therapy (mean therapy duration 5.8 ± 3.7 months) (Wucherpfennig et al., 2022a). Furthermore, MRI also showed improvements of the CRS-MRI sum score in adults with at least one F508del mutation and established disease after at least 1 month of therapy with elaxacaftor/tezacaftor/ivacaftor (Wucherpfennig et al., 2022b). However, the long-term effects of treatment with CFTRm on paranasal sinus abnormalities especially when initiated early in preschool age are unknown. Thus, the hypothesis of this longitudinal real-world observational study was, that the effects of first therapy with lumacaftor/ivacaftor on the development of paranasal sinuses, and the severity of paranasal sinus abnormalities are assessable by annual paranasal sinus MRI in conjunction with the CRS-MRI scoring system in 39 preschool and school-age children with CF over a median period of 3 years.

## Materials and methods

### Study population

This study was approved by the institutional ethics committee (clinicaltrials.gov identifier NCT02270476, S-211/2011, S-370/2011) and written informed consent was obtained from all parents or legal guardians. The diagnosis of CF was based on newborn screening (*n* = 9), clinical symptoms (*n* = 22) or both (*n* = 8), and confirmed by increased sweat Cl^−^ concentrations (≥60 mmol/L), *CFTR* mutation analysis (Hirtz et al., 2004; Graeber et al., 2022). 39 children with CF homozygous for F508del (mean age at baseline MRI 5.9 ± 3.0 years, range 1–12 years) who started lumacaftor/ivacaftor therapy at our center between February 2018 and December 2020 and underwent a paranasal sinus MRI before (MRI1) and at least one subsequent annual MRI (median of 3 examinations, range one to four MRI examinations) after first prescription of lumacaftor/ivacaftor (MRI2 to MRI5), were included (Table 1, Supplementary Figure S1). 15 children first started lumacaftor/ivacaftor in preschool age defined as an age-range of 1–5 years (mean 3.4 ± 1.0 years, range 2–5 years) and 24 children started lumacaftor/ivacaftor in school age defined as an age ≥6 years (mean 8.0 ± 2.0 years, range 6–12 years). MRI2 was performed on average 7.3 ± 4.1 months (range 1–16 months) after therapy initiation, while the interval between the consecutive annual MRI examinations was 12.5 ± 1.8 months (range 7–17 months). The mean observation time for children receiving lumacaftor/ivacaftor in preschool age was 31.6 ± 13.3 months and for children receiving lumacaftor/ivacaftor in school age 38.8 ± 12.2 months. Three examinations were aborted due to restlessness and could not be analysed. In total 143 MRI examinations were evaluated for this study. All children received additional symptomatic mucolytic therapy of the lower airways, seven received nasal saline irrigation and nine received nasal steroids. Patients who stopped lumacaftor/ivacaftor therapy due to change of CFTRm therapy regime (*n* = 22) or underwent surgery of the paranasal sinuses (*n* = 1) were excluded from this timepoint on. Cultures of upper airways were routinely obtained from nasal swabs as previously described (Supplementary Table S1) (Boutin et al., 2015; Sommerburg et al., 2020; Chung et al., 2021). Of note, some of the patients have been included with our previous reports, in which we did not assess long-term longitudinal paranasal sinus MRI abnormalities under lumacaftor/ivacaftor therapy (Stahl et al., 2017; Stahl et al., 2019; Sommerburg et al., 2020; Chung et al., 2021; Wucherpfennig et al., 2022a). 24 of them were included in our previous study on short-term effects of lumacaftor/ivacaftor therapy (Wucherpfennig et al., 2022a).

**TABLE 1:** Patient baseline characteristics. Data presented as mean ± standard deviation, and absolute and relative numbers, respectively. BMI, body mass index, *CFTR, cystic fibrosis transmembrane conductance regulator*, ppFEV1, percent predicted forced expiratory volume in 1 s, SDS, standard deviation score. ***p < 0.001 vs. lumacaftor/ivacaftor from preschool age.

	All	Lumacaftor/ivacaftor from preschool age	Lumacaftor/ivacaftor from school age
n =	39	15	24
Age (y)	5.9 ± 3.0	3.4 ± 1.0	8.0 ± 2.0
Sex (m/f)	19/20	7/8	12/12
Height (cm)	116.4 ± 21.5	69.7 ± 16.0	128.8 ± 13.9***
*Height, SDS*	−0.3 ± 1.2	−0.5 ± 1.1	−0.1 ± 1.2
Weight (kg)	21.9 ± 9.5	14.3 ± 4.3	26.7 ± 8.6***
*Weight, SDS*	−0.3 ± 0.9	−0.4 ± 0.6	−0.2 ± 1.0
BMI (kg/m^2^)	15.5 ± 1.6	15.2 ± 1.2	15.7 ± 1.7
*BMI, SDS*	−0.4 ± 0.7	−0.4 ± 0.7	−0.4 ± 0.7
*CFTR* genotype, n (%)			
*F508del/F508del*	39 (100)	15 (100)	24 (100)
Pancreatic insufficiency, n (%)	39 (100)	15 (100)	24 (100)
Spirometry, n (%)	32 (82)	8 (53)	24 (100)
*ppFEV1*	90.4 ± 15.5	88.0 ± 13.2	91.3 ± 16.3

### Magnetic resonance imaging

Standardized MRI of the paranasal sinuses was performed using the identical 1.5 T scanner and protocol (Magnetom Avanto, Siemens Healthcare, Erlangen, Germany) as previously described (Oltmanns et al., 2016; Sommerburg et al., 2020; Chung et al., 2021; Wucherpfennig et al., 2022a; Wucherpfennig et al., 2022b). In brief, T2-weighted sequences, and T1-weighted sequences before and after intravenous contrast application (Dotarem, Guerbet AG, Zurich, Switzerland; or Gadovist, Bayer AG, Leverkusen, Germany) were acquired. All subjects ≤5 years were routinely sedated with oral or rectal chloral hydrate (100 mg/kg body weight, maximum dose 2 g), and monitored by MRI-compatible pulse oximetry as previously described (Wielpütz et al., 2014; Stahl et al., 2019). The previously described CRS-MRI scoring system evaluates the maxillary, frontal, sphenoid and ethmoid sinus. The score comprises items rated for each sinus such as dimensions (length, width), degree of opacification (0 = none, 1 = less than 50%, 2 = 50%–99%, and 3 = complete opacification) and the specific abnormalities mucosal swelling, mucopyoceles, polyps and effusion (0 = none, 1 = present, and 2 = present and dominant), and deformation of the semilunar hiatus for the maxillary sinus only (0 = none, 1 = mucosal prolapse, and 2 = mucosal prolapse with contact to nasal septum). The maximal CRS-MRI sum score is 68 (Sommerburg et al., 2020; Chung et al., 2021; Wucherpfennig et al., 2022a; Wucherpfennig et al., 2022b). 60 of all 143 MRI examinations were assessed by two readers achieving almost perfect inter-reader agreement (Supplementary Table S2) (Sommerburg et al., 2020; Chung et al., 2021; Wucherpfennig et al., 2022a; Wucherpfennig et al., 2022b). Results from one reader are shown in the following analyses for all examinations. Due to low prevalence and differences between age groups of the frontal sinus in our cohort the frontal sinus subscore was excluded from the CRS-MRI sum score. For more information see the online data supplement.

### Statistical analyses

Data were analysed using Prism (version 9.1.0, GraphPad Software, San Diego, CA, United States) and are presented as mean ± standard deviation. For intra individual analysis for comparing two measurements Wilcoxon signed-rank test and for comparing more than two measurements ANOVA mixed-effects analysis including Geisser-Greenhouse correction, and for interindividual group analysis Mann-Whitney test and/or unpaired *t*-test were used. Prevalence and dominance were compared by Fisher’s exact test. A *p*-value <0.05 was considered statistically significant.

## Results

### Growth of paranasal sinuses in school-age and preschool children with CF is not changed under lumacaftor/ivacaftor therapy

In all children with CF, all maxillary, sphenoid and ethmoid sinuses were present (Supplementary Table S3). The frontal sinus was undetectable in all children who started lumacaftor/ivacaftor therapy in preschool age and was detectable in only 6% of children with CF starting therapy with lumacaftor/ivacaftor in school age (*p* = 0.085 vs children starting therapy in preschool age) (Supplementary Table S3).

**FIGURE 1:**
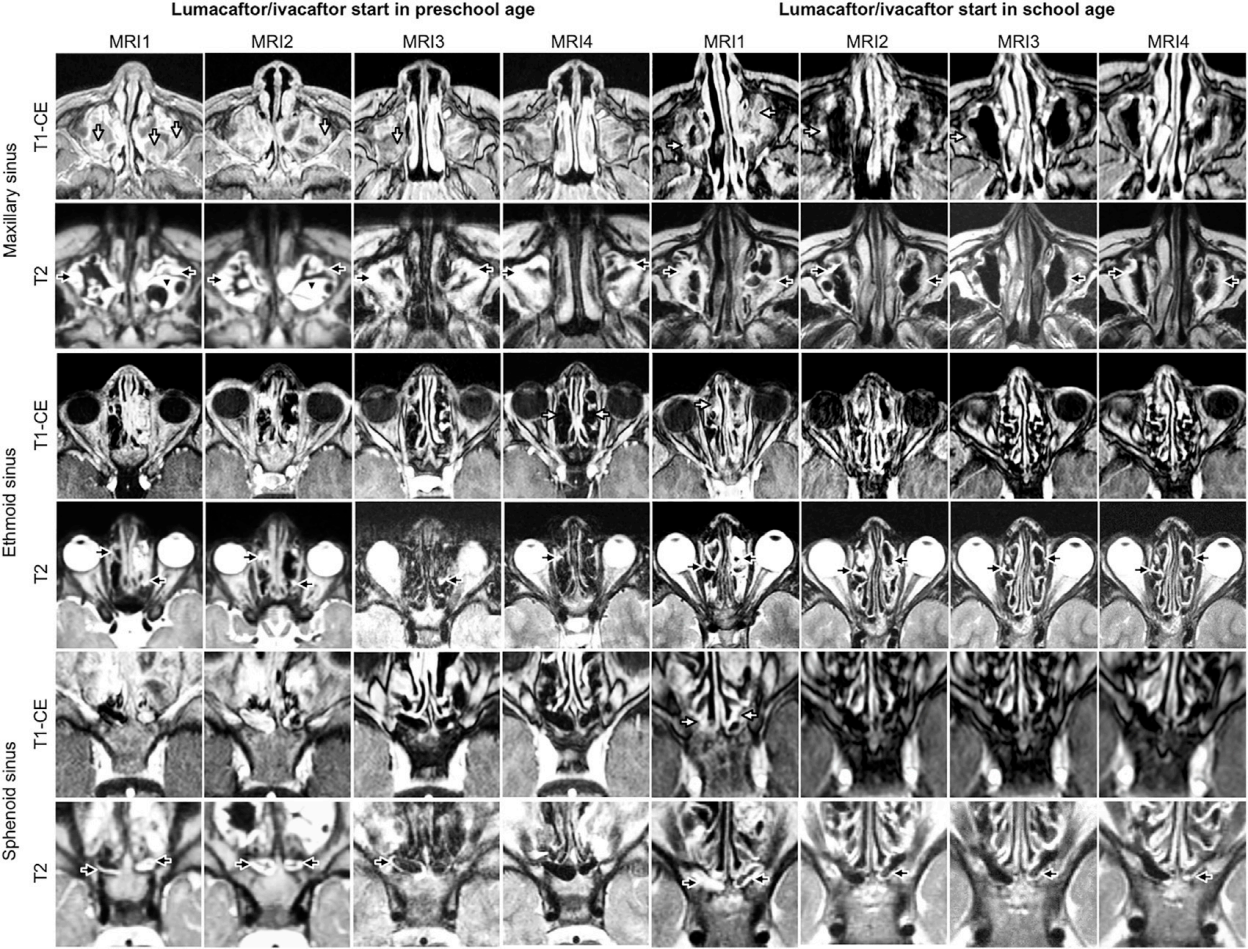
Representative examples of longitudinal magnetic resonance imaging of chronic rhinosinusitisparansal sinus abnormalities before (MRI1) and under therapy with lumacaftor/ivacaftor (MRI2-4) in children with cystic fibrosis starting lumacaftor/ivacaftor therapy in preschool and school age. Mucosal swelling is indicated by black arrows, mucopyoceles by white arrows and polyps by black arrowheads. Note the reduction of mucopyoceles in the maxillary sinus in the patient starting therapy in school age. The chronic rhinosinusitis magnetic resonance imaging (CRS-MRI) score in the patient starting therapy in preschool age (4 years at MRI1) was in MRI1 33, in MRI2 33, in MRI3 30 and in MRI4 29 and in the patient starting therapy in school age (9 years at MRI1) in MRI1 32, in MRI2 30, in MRI3 27 and in MRI4 30.

**FIGURE 2:**
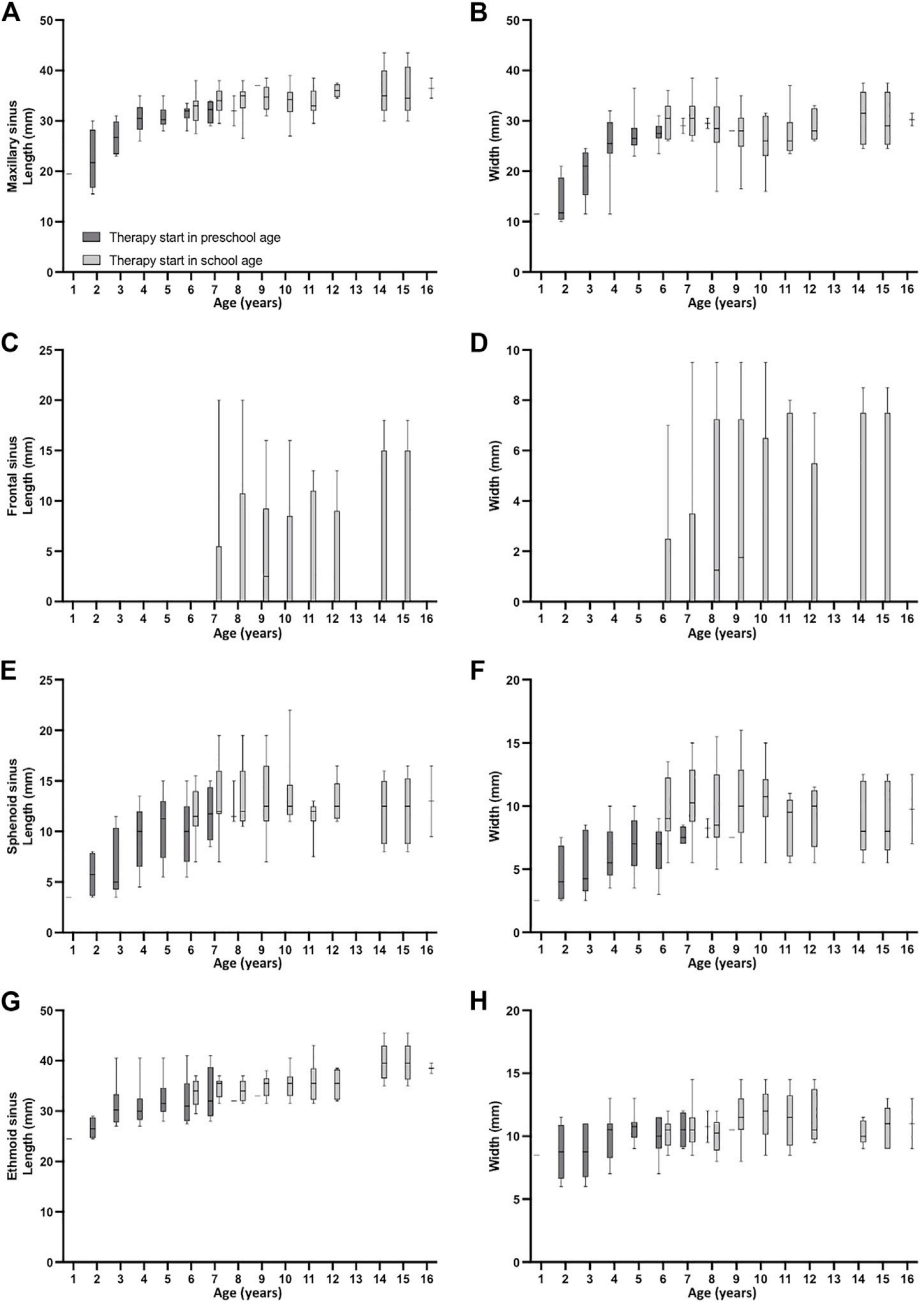
Development of paranasal sinus dimensions before (MRI1) and under therapy with lumacaftor/ivacaftor (MRI2-5) in children with cystic fibrosis starting lumacaftor/ivacaftor therapy in preschool and school age. Measurements of the dimensions of the maxillary **(A, B)**, frontal **(C, D)**, sphenoid **(E, F)** and ethmoid **(G, H)** sinus. Both sides were averaged per patient. Boxes represent 25th to 75th percentile, the median is indicated by a horizontal line, and whiskers mark 5th and 95th percentiles. Spearman correlation coefficients r with *p*-values for dimensions vs. age are indicated in each panel.

On average, on MRI1 the sinus dimensions in children starting lumacaftor/ivacaftor in school age were larger than in the group starting lumacaftor/ivacaftor in preschool age (*p* < 0.05), except for width of ethmoid sinus (*p* = 0.181). In preschool age, the growth of paranasal sinuses was more pronounced than in school age (Figures 1, 2). Overall, the growth curves of sinus dimensions of children starting therapy in preschool age harmoniously blend into the sinus dimensions at baseline MRI1 from children starting therapy at school age (Figure 2), which together with our previous longitudinal report without CFTRm therapy indicates that lumacaftor/ivacaftor did not affect growth rates (Wucherpfennig et al., 2022a).

### Lumacaftor/ivacaftor therapy decreases chronic rhinosinusitis severity in school-age children with CF

**FIGURE 3:**
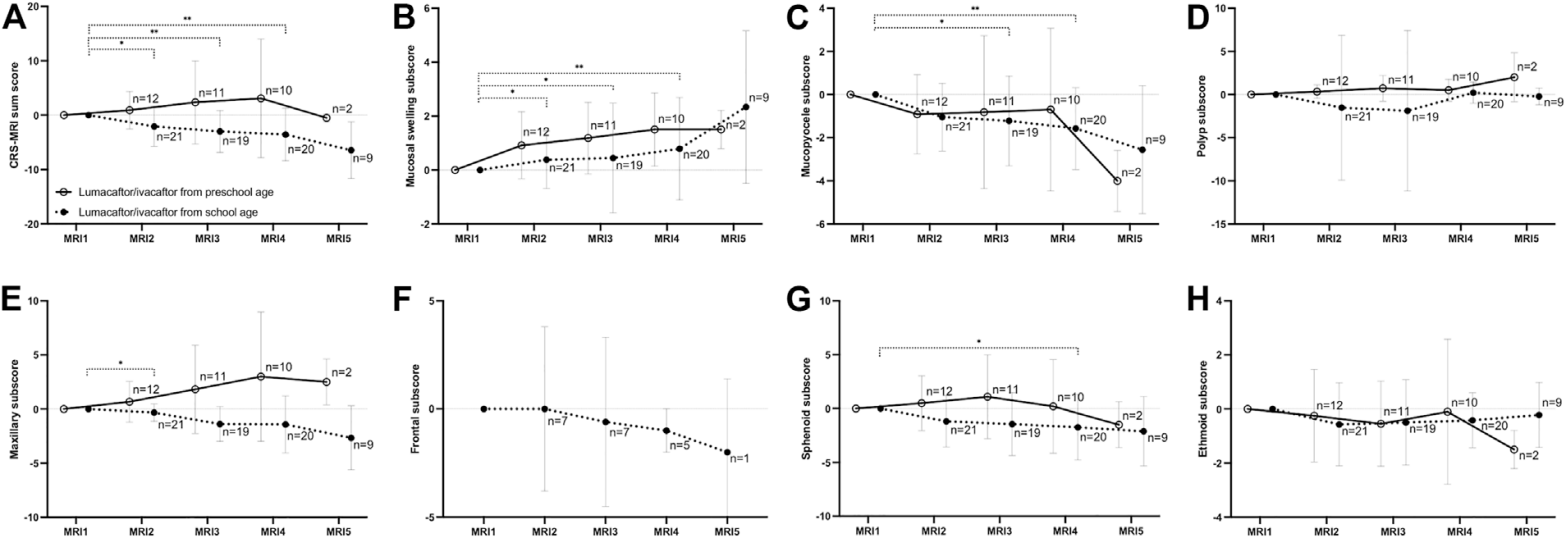
Changes of the chronic rhinosinusitis magnetic resonance imaging (CRS-MRI) sum score and subscores from baseline (MRI1) after therapy start with lumacaftor/ivacaftor (MRI2-5) in children with cystic fibrosis. The CRS-MRI sum score **(A)**, abnormality subscores **(B–D)** and sinus subscores **(E–H)** were grouped by MRI timepoints. The mean is indicated by a circle and whiskers mark the standard deviation. *p < 0.05 vs. MRI1, **p < 0.01 vs. MRI1.

In children starting lumacaftor/ivacaftor therapy in school age, the prevalence of opacified sphenoid sinuses decreased in MRI3 and MRI4 compared to MRI1 (84% and 85% vs. 100%, respectively; *p* < 0.01 vs. MRI1). In the maxillary sinus, the prevalence of mucopyoceles decreased in MRI4 and MRI5 compared to MR1 (75% and 72% vs. 92%, respectively; *p* < 0.05 vs. MRI1). Moreover, the dominance of mucopyoceles decreased especially in MRI4 and MRI5 compared to MRI1 in the maxillary sinus (25% and 17% vs. 55%, respectively; *p* < 0.01). Also, in ethmoid sinus the dominance of mucopyoceles decreased in MRI3 and MRI4 (16% and 20% vs. 33%, *p* < 0.05) (Figure 1; Table 2; Supplementary Table S4–S6). This leads to a decrease in the mucopyocele subscore in MRI3 and MRI4 compared to MRI1 (−1.2 ± 2.0 and −1.6 ± 1.8, respectively; *p* < 0.05), and conversely in an increase in mucosal swelling subscore in MRI2, MRI3, and MRI4 compared to MRI1 (*p* < 0.05). Moreover, the maxillary sinus subscore decreased from MRI1 to MRI2 and sphenoid subscore from MRI1 to MRI4 (*p* < 0.05). The CRS-MRI sum score decreased longitudinally from MRI1 to MRI2, MRI3, and MRI4 (−2.1 ± 3.5, −3.0 ± 3.7 and −3.6 ± 4.7, respectively; *p* < 0.05–0.01) (Figure 3, Supplementary Figure S2).

**TABLE 2:** Chronic rhinosinusitis magnetic resonance imaging (CRS-MRI) scores for the maxillary sinus in children with cystic fibrosis before (MRI1) and after start of lumacaftor/ivacaftor therapy (MRI2-5) in preschool age or school age. Prevalence n (%) and dominance n (%) of sinus abnormalities are presented on a per-sinus basis, and sinus subscore as mean ± standard deviation. *p < 0.05 vs. MRI1, **p < 0.01 vs. MRI1. For the frontal, sphenoid and ethmoid sinus please refer to Supplementary Table S4–S6.

			MRI1	MRI2	MRI3	MRI4	MRI5
Lumacaftor/ivacaftor from preschool age	**Maxillary sinus,** n		30	24	22	20	4
	**Opacification**	Prevalence, n (%)	26 (87)	23 (96)	20 (91)	20 (100)	4 (100)
	**Mucosal swelling**	Prevalence, n (%)	26 (87)	23 (96)	20 (91)	20 (100)	4 (100)
		Dominance, n (%)	2 (8)	4 (17)	3 (15)	7 (35)*	1 (25)
	**Mucopyoceles**	Prevalence, n (%)	25 (83)	19 (79)	20 (91)	17 (85)	4 (100)
		Dominance, n (%)	17 (65)	12 (52)	9 (45)	9 (45)	1 (25)
	**Polyps**	Prevalence, n (%)	20 (67)	18 (75)	16 (73)	14 (70)	4 (100)
		Dominance, n (%)	6 (23)	6 (26)	7 (35)	3 (15)	2 (50)
	**Effusion**	Prevalence, n (%)	0 (0)	0 (0)	0 (0)	0 (0)	0 (0)
		Dominance, n (%)	0 (0)	0 (0)	0 (0)	0 (0)	0 (0)
	**Deformation of semilunar hiatus**	Prevalence, n (%)	25 (83)	20 (83)	17 (77)	19 (95)	4 (100)
	**Maxillary sinus subscore**		**14.1 ± 5.6**	**14.1 ± 5.7**	**13.7 ± 6.4**	**15.6 ± 2.5**	**17.0 ± 1.0**
Lumacaftor/ivacaftor from school age	**Maxillary sinus,** n		48	42	38	40	18
	**Opacification**	Prevalence, n (%)	47 (98)	41 (98)	37 (97)	40 (100)	18 (100)
	**Mucosal swelling**	Prevalence, n (%)	47 (98)	41 (98)	37 (97)	40 (100)	18 (100)
		Dominance, n (%)	12 (26)	15 (37)	16 (43)	21 (53)*	11 (61)*
	**Mucopyoceles**	Prevalence, n (%)	44 (92)	38 (90)	30 (79)	30 (75)*	13 (72)*
		Dominance, n (%)	26 (55)	15 (37)	10 (27)*	10 (25)**	3 (17)**
	**Polyps**	Prevalence, n (%)	28 (58)	25 (60)	15 (66)	24 (60)	9 (50)
		Dominance, n (%)	9 (19)	11 (27)	11 (30)	9 (23)	4 (22)
	**Effusion**	Prevalence, n (%)	0 (0)	0 (0)	0 (0)	0 (0)	0 (0)
		Dominance, n (%)	0 (0)	0 (0)	0 (0)	0 (0)	0 (0)
	**Deformation of semilunar hiatus**	Prevalence, n (%)	46 (96)	40 (95)	32 (84)	35 (88)	18 (100)
	**Maxillary sinus subscore**		**15.3 ± 3.3**	**14.7 ± 3.4**	14.2 ± 3.8**	13.9 ± 4.0**	14.0 ± 3.6*

The bold values are the sinus subscores.

### Lumacaftor/ivacaftor therapy stabilizes disease progression of chronic rhinosinusitis severity in preschool children with CF

The CRS-MRI sum score was similar in preschool and school-age children with CF at MRI1 (32.9 ± 7.8 vs. 34.6 ± 5.2, *p* = 0.847) (Figures 1, 3, Supplementary Figure S2). Baseline CRS-MRI sum score was similar for children diagnosed by newborn screening without symptoms compared to those diagnosed symptomatic (*p* = 0.152) and for children positive or negative for staphyloccocus aureus in nasal swabs (*p* = 0.561) (Supplementary Figure S2). The prevalence of germs detected by nasal swabs did not differ between the groups (*p* = 0.554–0.999).

In children starting lumacaftor/ivacaftor therapy in preschool age, the prevalence of opacified sinuses and prevalence of the abnormalities remained stable during therapy. The dominance of mucopyoceles in the ethmoid sinus decreased from MRI1 to MRI3 (43% vs. 14%, *p* < 0.05) (Table 2, Supplementary Table S4–S6). The mucopyocele subscore (−1.0 ± 1.8, −0.9 ± 3.5, −0.9 ± 3.5 and −4.0 ± 1.0, respectively; *p* = 0.411–0.774) and the mucosal swelling subscore did not change during therapy from MRI1 to MRI2, MRI3, MRI4 and MRI5 (0.8 ± 1.2, 1.3 ± 1.3, 1.2 ± 1.0 and 1.5 ± 0.5, respectively; *p* = 0.222–0.942 vs. MRI1) (Figure 3, Supplementary Figure S2). The CRS-MRI sum score and the sinus subscores remained stable during therapy (*p* = 0.520–0.999 vs. MRI1) (Figure 3, Supplementary Figure S2).

## Discussion

This is the first study assessing the long-term effects of treatment with lumacaftor/ivacaftor on prevalence and severity of paranasal sinus abnormalities in preschool and school-age children with CF by consecutive annual paranasal sinus MRI over a median period of 3 years. We demonstrate that in school-age children with CF homozygous for the F508del mutation severity of paranasal sinus abnormalities improves in response to therapy with lumacaftor/ivacaftor as detected by paranasal sinus MRI employing the CRS-MRI sum score. Moreover, progression of paranasal sinus abnormalities is prevented in children with CF homozygous for the F508del mutation by starting lumacaftor/ivacaftor therapy in preschool age (Figure 3; Table 2; Supplementary Table S4–S6).

In our study all maxillary, ethmoid and sphenoid sinuses were present, while the frontal sinus was present in only 6% of children with CF starting therapy with lumacaftor/ivacaftor in school age, and was undetectable in all children who started lumacaftor/ivacaftor therapy in preschool age (Supplementary Table S3). Previous CT and MRI studies similarly reported the earliest pneumatization of frontal sinus from the third year of life and a high degree of sinus aplasia in patients with CF (Kim et al., 1997; Spaeth et al., 1997; Shah et al., 2003; Sommerburg et al., 2020). Paranasal sinus dimensions in preschool and school-age children under lumacaftor/ivacaftor therapy were mostly in line with previous MRI studies assessing the development and abnormalities of paranasal sinuses in children with CF from infancy to school age without CFTRm (Sommerburg et al., 2020; Wucherpfennig et al., 2022a). Also, the growth curves of paranasal sinus dimensions under lumacaftor/ivacaftor therapy are comparable to previous longitudinal results in children without CFTRm, demonstrating a higher growth rate of paranasal sinuses in preschool than in school-age (Sommerburg et al., 2020; Wucherpfennig et al., 2022a). Further it was shown that sinus dimensions in children with CF aged between zero and 6 years are similar to sinus dimensions in a healthy control group of the same age (Sommerburg et al., 2020). This indicates in conjunction with the growth curves obtained from our previous study in children with CF without any CFTRm, that lumacaftor/ivacaftor has no effect on paranasal sinus development (Wucherpfennig et al., 2022a). Taken together, these results indicate that sinus growth does not seem to be influenced by presence and severity of paranasal sinus abnormalities nor by therapy with lumacaftor/ivacaftor.

In our cohort, the prevalence of opacified sinuses at baseline MRI1 in preschool and school-age children was in line with our previous reports (Figure 1; Table 2; Supplementary Table S4–S6) (Sommerburg et al., 2020; Wucherpfennig et al., 2022a). Moreover, baseline prevalence and dominance of the different abnormalities as well as sinus and abnormality subscores and the CRS-MRI sum score in the present study are comparable to the previous cross-sectional and the longitudinal cohort study assessing the onset and progression of paranasal sinus abnormalities in children without CFTRm therapy (Wucherpfennig et al., 2022a).

Our present study demonstrates that in children starting lumacaftor/ivacaftor therapy in school age, the prevalence of opacified sphenoid sinuses decreases longitudinally. Moreover, the prevalence and dominance of mucopyoceles decrease, especially in the maxillary sinus, leading to a decrease in mucopyoceles subscore as well as maxillary subscore (Figure 3; Table 2, Supplementary Table S4–S6). In addition, the CRS-MRI score improved in children with CF starting lumacaftor/ivacaftor therapy in school age from the first MRI examination, with subtle further improvements thereafter (Figure 3). The improvements in the CRS-MRI sum score after start of lumacaftor/ivacaftor in school-age children by an average of 2.1 score points in the first year of therapy is numerically higher than in the previous mid-term follow up MRI study in a group encompassing preschool and school-age children homozygous for F508del, which showed an improvement of the CRS-MRI sum score by 0.5 points after at least 1 month of therapy with lumacaftor/ivacaftor (Wucherpfennig et al., 2022a). Of note, the magnitude of improvements in the CRS-MRI sum score in our present study is lower than in a previous study on highly effective CFTRm therapy with elexacaftor/tezacaftor/ivacaftor, which has shown a reduction of the CRS-MRI score in adults with CF by 6.9 points (Wucherpfennig et al., 2022b). Since it was shown, that in patients with CF aged between twelve and 60 years improvements in paranasal sinus abnormalities at CT were accompanied by improvements in SNOT-22 within the first month of therapy with elexacaftor/tezacaftor/ivacaftor, it is reasonable that improvements in paranasal sinus abnormalities detected by imaging reflect improvements in CRS (Stapleton et al., 2022). Elexacaftor/tezacaftor/ivacaftor was shown to be more effective on lung function in adults with CF compared to lumacaftor/ivacaftor therapy and also on morpho-functional lung MRI using a semiquantitative CF score (Wainwright et al., 2015; Graeber et al., 2018; Heijerman et al., 2019; Middleton et al., 2019; Mall et al., 2020; Graeber et al., 2021; Nichols et al., 2021; Wucherpfennig et al., 2022b; Graeber et al., 2022). Therefore, it is conceivable that the differences in improvements of CRS-MRI sum scores are caused by the different therapy effectiveness. Conversely, this demonstrates a high sensitivity of paranasal sinus MRI and the CRS-MRI scoring system even for less effective therapeutic strategies.

Further, our present study demonstrates that in children starting lumacaftor/ivacaftor therapy in preschool age, the prevalence of opacified sinuses as well as the prevalence of the different abnormalities remained stable during therapy, while mucopyoceles were less dominant in ethmoid sinuses after therapy start (Table 2, Supplementary Table S4–S6). The CRS-MRI score remained stable over a period of a median of three annual follow-up MRI examinations (Figure 3, Supplementary Figure S2). Of note, this result reconciles the higher improvements found in the CRS-MRI sum score in school-age children in the present study with our previous report in a cohort consisting of preschool and school-age children (Wucherpfennig et al., 2022a). The inclusion of preschool children in the previous study could explain the lower numerical improvements found as compared to the present cohort of children starting lumacaftor/ivacaftor at school age. This result also needs to be compared against our previous longitudinal study, which demonstrated an increase in the CRS-MRI sum score, in maxillary and sphenoid subscores as well as in mucopyoceles subscore especially from infancy and during the early preschool period (0–3 years) (Wucherpfennig et al., 2022a). The reduction of dominance of mucopyoceles under lumacaftor/ivacaftor therapy in preschool children thus compares beneficially against the reported worsening without CFTRm treatment (Wucherpfennig et al., 2022a). Mucopyoceles are a pathognomonic feature of paranasal sinus abnormalities in CF, and might cause complications such as headache, bone resorption with consequent spread of infection (Wagenmann and Naclerio, 1992; Zukin et al., 2017). When symptomatic, mucopyoceles are usually decompressed by endoscopic surgery (Wagenmann and Naclerio, 1992; Zukin et al., 2017). The indication for surgery is mainly based on clinical symptoms, which we could not assess in our study (Rasmussen et al., 2012). In the absence of a control group in the present study, our data may be compared against previously published results on a longitudinal cohort of children with CF, which has shown an increase in the CRS-MRI score from infancy to school-age (Wucherpfennig et al., 2022a). By comparison, lumacaftor/ivacaftor therapy starting in preschool age could be able to prevent the progression of paranasal sinus abnormalities in the sense of a preventive treatment.

Both, lumacaftor/ivacaftor and also elexacaftor/tezacaftor/ivacaftor therapy lead to a reduction in mucopyoceles especially in the maxillary sinus (Wucherpfennig et al., 2022a; Wucherpfennig et al., 2022b). The reduction of mucopyoceles was paralleled by an increase in dominance of mucosal swelling and consequently an increase in the mucosal swelling subscore. This is due to rules of the CRS-MRI scoring system, by which an opacified sinus always is assigned one dominant abnormality. Therefore, the increase in the mucosal swelling subscore is not necessarily associated with an actual increase in the severity of mucosal swelling itself but it is inverse to the reduction in prevalence and dominance of mucopyoceles.

Our study has the limitation, that we did not obtain a CRS symptom score such as SNOT-22 to systematically assess the relationship between the various structural abnormalities and inflammation or clinical disease burden. The improvements in the CRS-MRI sum score are very likely related to improvements in the sinonasal outcome test, though there has been no direct comparison yet between MRI and clinical severity (DiMango et al., 2021; Beswick et al., 2022). Further, the clinical significance of a reduction of mucopyoceles for the regional microbiome remains to be studied. Moreover, our study was performed in absence of a control group. Therefore, we compared our data may against previously published results on a longitudinal cohort of children with CF, which has shown an increase in the CRS-MRI score from infancy to school-age (Wucherpfennig et al., 2022a).

In conclusion, our study demonstrates a positive long-term effect of therapy initiation with lumacaftor/ivacaftor on paranasal sinus abnormalities in school-age and preschool children over a median period of 3 years in two ways: 1) Improvements in the CRS-MRI sum score after therapy initiation with lumacaftor/ivacaftor in school-age children with CF. 2) Prevention of an increase in the CRS-MRI sum score by therapy initiation in preschool children with CF as opposed to the previously shown worsening with natural disease progression. The observed differences in response to lumacaftor/ivacaftor therapy in school-age vs. preschool children support the concept that an early initiation of targeted therapy may be most effective in delaying or even preventing paranasal sinus abnormalities. Further, we demonstrate the potential of annual comprehensive paranasal sinus MRI as a sensitive non-invasive and radiation-free diagnostic tool for the monitoring of disease progression and therapy response of CF-related abnormalities of the upper airway tract from early childhood.

## Data Availability

The raw data supporting the conclusion of this article will be made available by the authors, without undue reservation.
