# Supplementary material for: Long-term effects of lumacaftor/ivacaftor on paranasal sinus abnormalities in children with cystic fibrosis detected with magnetic resonance imaging
Source: Front Pharmacol. 2023 Apr 10;14:1161891. doi: 10.3389/fphar.2023.1161891 (PMC10123276; doi:10.3389/fphar.2023.1161891)
Supplement: Supplementary file 1 [file DataSheet1.docx]

**Long-Term Effects of Lumacaftor/Ivacaftor on Paransal Sinus Abnormalities in Children with Cystic Fibrosis Detected with Magnetic Resonance Imaging**

Lena Wucherpfennig, Felix Wuennemann, Monika Eichinger, Angelika Seitz, Ingo Baumann, Mirjam Stahl, Simon Y. Graeber, Shengkai Zhao, Jaehi Chung, Jens-Peter Schenk, Abdulsattar Alrajab, Hans-Ulrich Kauczor, Marcus A. Mall, Olaf Sommerburg, Mark O. Wielpütz

*Online Data Supplement*

**SUPPLEMENTAL METHODS**

**Magnetic resonance imaging**

MRI of paranasal sinuses was performed on a clinical 1.5T scanner (Magnetom Avanto, Siemens Healthineers, Erlangen, Germany) using a head coil. All subjects ≤5 years were routinely sedated with oral or rectal chloral hydrate (100 mg/kg body weight, maximum dose of 2 g) and monitored by MRI compatible pulse oximetry as previously described (E1, E2). T1-weighted images were obtained with a three-dimensional gradient echo sequence in axial plane with 0.4 mm^2^ in-plane resolution and 1.0 mm slice thickness (VIBE) before and after intravenous administration of gadolinium-based contrast (Dotarem, Guerbet AG, Zurich, Switzerland; or Gadovist, Bayer AG, Germany). T2-weighted images were obtained in sagittal plane with 0.5-1.0 mm^2^ in-plane resolution and 1.0 mm slice thickness (SPACE). All acquisitions were reconstructed in three orthogonal planes. The total examination time is about 10 minutes. Note that in infants contrast material was not routinely used due to applicable prescription restrictions (n=4).

All MRI examinations were assessed by two readers with more than three years of experience in MRI of CF using the previously described chronic rhinosinusitis (CRS)-MRI score (LW). 60 randomly chosen MRI examinations were additionally assessed by one reader with more than six years of experience in MRI of CF using the chronic rhinosinusitis (CRS)-MRI score (FW) for inter-reader agreement. The CRS-MRI score evaluates the maxillary, frontal, sphenoid and ethmoid sinus. Maximal long and perpendicular short-axis diameters were measured manually for the maxillary, frontal, sphenoid, and ethmoid sinus on axial images on both sides. Furthermore, the degree of opacification of each sinus was assessed on a four-point scale with 0 = none, 1 = less than 50%, 2 = 50% to 99%, and 3 = complete opacification. If a score >0 was assigned, the sinus was further evaluated for mucosal swelling, mucopyoceles, polyps, and effusion using a three-point scale: 0 = none, 1 = present, and 2 = present and dominant. A score of 2 could be selected only once per sinus and side, whereas a score of 1 could be assigned to multiple abnormalities. For the maxillary sinus, deformation of the semilunar hiatus was additionally assessed on a three-point scale: 0 = none, 1 = mucosal prolapse, and 2 = mucosal prolapse with contact to nasal septum. The maximal CRS-MRI score is 68 (E3, E4). Measurements were carried out in the institutional PACS system on a certified workstation and screens.

**SUPPLEMENTAL RESULTS**

**Chronic rhinosinusitis magnetic resonance imaging (CRS-MRI) scoring system shows almost perfect inter-reader agreement**

Inter-reader agreement for the CRS-MRI score was assessed by Cohens weighted kappa (κ) for the CRS-MRI sum score as well as for subscores for each sinus. The CRS-MRI score showed an almost perfect agreement for both readers with κ=0.922 for the CRS-MRI sum score, as well as κ=0.834-0.952 for the sinus subscores (Supplemental Table E2). Because inter-reader agreement was found to be almost perfect and calculation of score means is impracticable (compare item ‘dominance’), the scores of one reader (LW) are presented in the main manuscript as in our previous study (E3).

**SUPPLEMENTAL REFERENCES**

E1. Wielpütz MO, Puderbach M, Kopp-Schneider A, Stahl M, Fritzsching E,

Sommerburg O, Ley S, Sumkauskaite M, Biederer J, Kauczor HU, Eichinger M, Mall MA. Magnetic resonance imaging detects changes in structure and perfusion, and response to therapy in early cystic fibrosis lung disease. Am J Respir Crit Care Med 2014; 189: 956-965.

E2. Stahl M, Wielpütz MO, Ricklefs I, Dopfer C, Barth S, Schlegtendal A, Graeber SY, Sommerburg O, Diekmann G, Husing J, Koerner-Rettberg C, Nahrlich L, Dittrich AM, Kopp MV, Mall MA. Preventive Inhalation of Hypertonic Saline in Infants with Cystic Fibrosis (PRESIS). A Randomized, Double-Blind, Controlled Study. Am J Respir Crit Care Med 2019; 199: 1238-1248.

E3. Sommerburg O, Wielpütz MO, Trame JP, Wuennemann F, Opdazaite E, Stahl M, Puderbach MU, Kopp-Schneider A, Fritzsching E, Kauczor HU, Baumann I, Mall MA, Eichinger M. Magnetic Resonance Imaging Detects Chronic Rhinosinusitis in Infants and Preschool Children with Cystic Fibrosis. Ann Am Thorac Soc 2020; 17: 714-723.

E4. Chung J, Wünnemann F, Salomon J, Boutin S, Frey DL, Albrecht T, Joachim C, Eichinger M, Mall MA, Wielpütz MO, Sommerburg O. Increased Inflammatory Markers Detected in Nasal Lavage Correlate with Paranasal Sinus Abnormalities at MRI in Adolescent Patients with Cystic Fibrosis. Antioxidants (Basel) 2021; 10.

**SUPPLEMENTAL FIGURES**

**
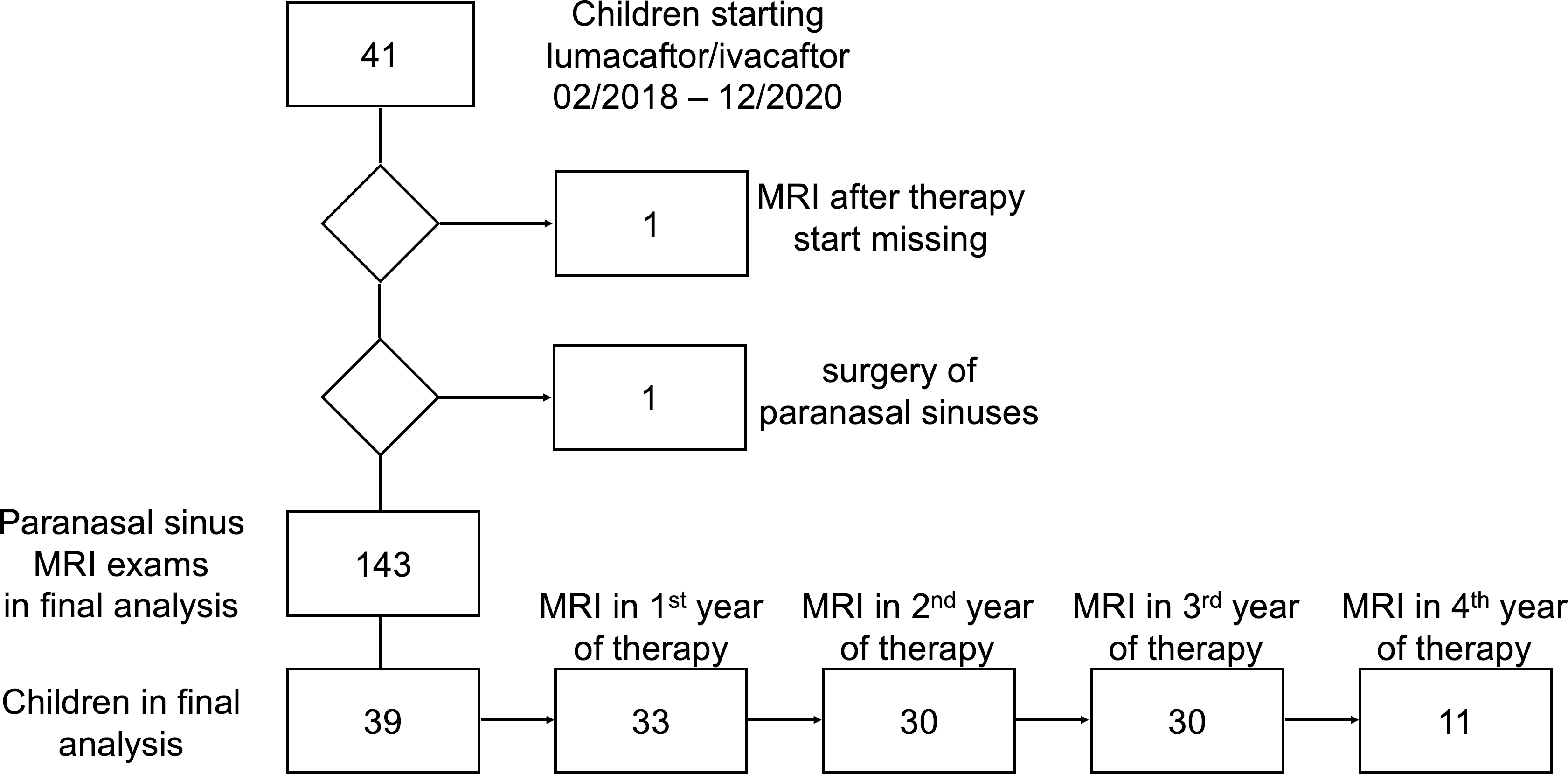
**

**Supplemental Figure E1. Study flowchart.**

**
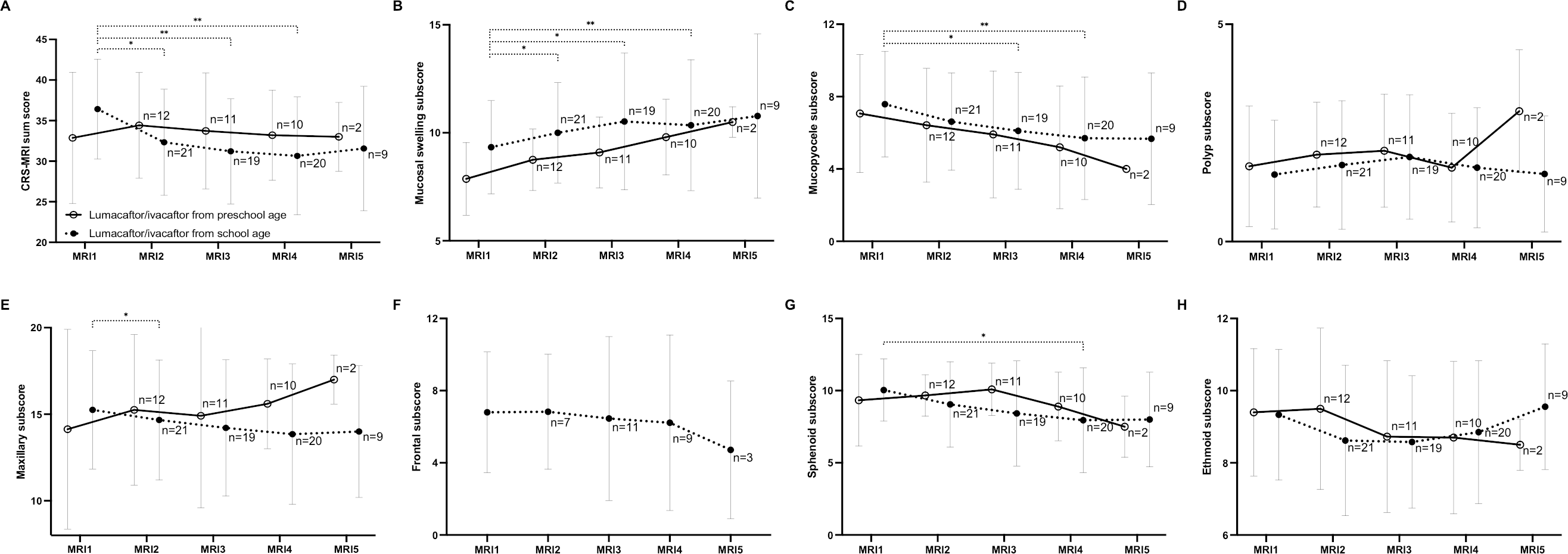
**

**Supplemental Figure E2. Longitudinal development of the chronic rhinosinusitis magnetic resonance imaging (CRS-MRI) sum score and subscores before (MRI1) and annually under (MRI2 to MRI5) therapy with lumacaftor/ivacaftor in children with cystic fibrosis.** The CRS-MRI sum score (A), abnormality subscores (B-D) and sinus subscores (E-H) were grouped by MRI timepoints. The mean is indicated by a line and whiskers mark the standard deviation. *p<0.05 vs. MRI1, **p<0.01 vs. MRI1.


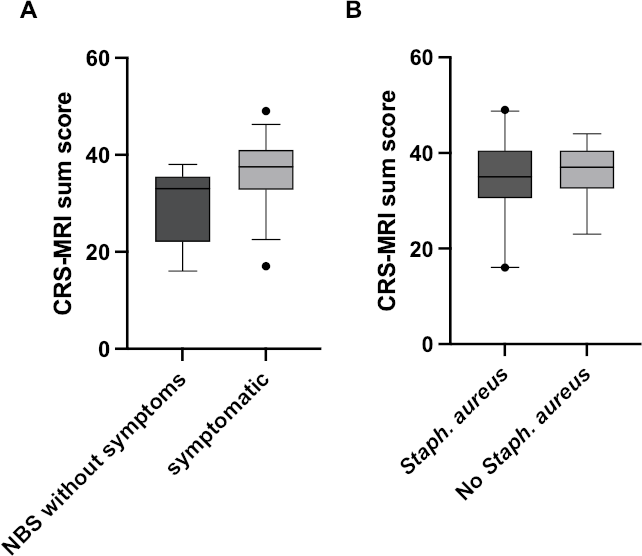


**Supplemental Figure E3. Baseline chronic rhinosinusitis magnetic resonance imaging (CRS-MRI) scores in children with cystic fibrosis depending on diagnosis based on newborn screening (NBS) without symptoms or symptoms (with/without NBS) (A) and Staphylococcus aureus infection status (B).** Boxes represent 25^th^ to 75^th^ percentile, the median is indicated by a horizontal line, and whiskers mark 5^th^ and 95^th^ percentiles. Circles represent outliers. Staph. = Staphylococcus.

**SUPPLEMENTAL TABLES**

|  | ***Pseudomonas aeruginosa*** | **Staphylococcus aureus** | ***Haemophilus influenzae*** | ***Moraxella catarrhalis*** | ***Streptococcus pneumoniae*** | ***Aspergillus fumigatus*** | ***Candida albicans*** | **Coagulase-negative Staphylococcus** | **Corynebacterium species** |
| --- | --- | --- | --- | --- | --- | --- | --- | --- | --- |
| **Subjects with nasal swab, n** | 37 | 37 | 37 | 37 | 37 | 37 | 37 | 37 | 37 |
| **Prevalence, n (%)** | 0 (0) | 17 (46) | 3 (8) | 2 (5) | 0 (0) | 0 (0) | 1 (3) | 14 (38) | 9 (24) |

**Supplemental Table E1.** **Patient baseline nasal bacterial colonization.** Data presented in absolute and relative numbers.

|  | **Agreement, κ** |
| --- | --- |
| **Maxillary sinus subscore** | 0.855 (0.733-0.976) |
| **Frontal sinus subscore** | 0.952 (0.862-1.042) |
| **Sphenoid sinus subscore** | 0.834 (0.680-0.989) |
| **Ethmoid sinus subscore** | 0.834 (0.688-0.979) |
| **CRS-MRI sum score** | 0.922 (0.884-0.960) |

**Supplemental Table E2. Inter-reader agreement for the chronic rhinosinusitis magnetic resonance imaging (CRS-MRI) scoring system in children with cystic fibrosis.** Cohen's weighted κ and 95% confidence intervals are given.

|  |  | **MRI1** | **MRI2** | **MRI3** | **MRI4** | **MRI5** |
| --- | --- | --- | --- | --- | --- | --- |
| **Lumacaftor/ivacaftor from preschool age** | **Subjects,** n | 15 | 12 | 11 | 10 | 2 |
|  | **Maxillary sinus,** n (%) | 15 (100) | 12 (100) | 11 (100) | 10 (100) | 2 (100) |
|  | **Frontal sinus,** n (%) | 0 (0) | 0 (0) | 0 (0) | 0 (0) | 0 (0) |
|  | **Sphenoid sinus,** n (%) | 15 (100) | 12 (100) | 11 (100) | 10 (100) | 2 (100) |
|  | **Ethmoid sinus,** n (%) | 15 (100) | 12 (100) | 11 (100) | 10 (100) | 2 (100) |
| **Lumacaftor/ivacaftor from school age** | **Subjects,** n | 24 | 21 | 19 | 20 | 9 |
|  | **Maxillary sinus,** n (%) | 24 (100) | 21 (100) | 19 (100) | 20 (100) | 9 (100) |
|  | **Frontal sinus,** n (%) | 7 (29) | 7 (33) | 11 (58) | 9 (45) | 3 (33) |
|  | **Sphenoid sinus,** n (%) | 24 (100) | 21 (100) | 19 (100) | 20 (100) | 9 (100) |
|  | **Ethmoid sinus,** n (%) | 24 (100) | 21 (100) | 19 (100) | 20 (100) | 9 (100) |

**Supplemental Table E3. Presence of paranasal sinuses in children with cystic fibrosis before (MRI1) and after start of lumacaftor/ivacaftor therapy (MRI2-5) in preschool age or school age.** Sinus presence (at least one side present) given on a per-patient basis.

|  |  |  | MRI1 | MRI2 | MRI3 | MRI4 | MRI5 |
| --- | --- | --- | --- | --- | --- | --- | --- |
| Lumacaftor/ivacaftor from preschool age |  | **Frontal sinus,** n | 0 | 0 | 0 | 0 | 0 |
|  | **Opacification** | Prevalence, n (%) | - | - | - | - | - |
|  | **Mucosal swelling** | Prevalence, n (%) | - | - | - | - | - |
|  |  | Dominance, n (%) | - | - | - | - | - |
|  | **Mucopyoceles** | Prevalence, n (%) | - | - | - | - | - |
|  |  | Dominance, n (%) | - | - | - | - | - |
|  | **Polyps** | Prevalence, n (%) | - | - | - | - | - |
|  |  | Dominance, n (%) | - | - | - | - | - |
|  | **Effusion** | Prevalence, n (%) | - | - | - | - | - |
|  |  | Dominance, n (%) | - | - | - | - | - |
|  | **Frontal sinus subscore** | | - | - | - | - | - |
| Lumacaftor/ivacaftor from school age |  | **Frontal sinus,** n | 11 | 13 | 19 | 15 | 5 |
|  | **Opacification** | Prevalence, n (%) | 8 (73) | 11 (85) | 14 (74) | 11 (73) | 5 (100) |
|  | **Mucosal swelling** | Prevalence, n (%) | 8 (73) | 11 (85) | 14 (74) | 11 (73) | 5 (100) |
|  |  | Dominance, n (%) | 7 (64) | 8 (73) | 11 (79) | 9 (82) | 4 (80) |
|  | **Mucopyoceles** | Prevalence, n (%) | 4 (36) | 7 (54) | 7 (54) | 9 (60) | 4 (80) |
|  |  | Dominance, n (%) | 2 (29) | 3 (27) | 3 (21) | 2 (18) | 1 (20) |
|  | **Polyps** | Prevalence, n (%) | 0 (0) | 1 (8) | 1 (5) | 1 (7) | 1 (20) |
|  |  | Dominance, n (%) | 0 (0) | 0 (0) | 0 (0) | 0 (0) | 0 (0) |
|  | **Effusion** | Prevalence, n (%) | 0 (0) | 0 (0) | 0 (0) | 0 (0) | 0 (0) |
|  |  | Dominance, n (%) | 0 (0) | 0 (0) | 0 (0) | 0 (0) | 0 (0) |
|  | **Frontal sinus subscore** | | **6.8±3.0** | **6.8±2.9** | **6.5±4.3** | **6.2±4.6** | **6.0±4.1** |

**Supplemental Table E4. Chronic rhinosinusitis magnetic resonance imaging (CRS-MRI) scores for the frontal sinus in children with cystic fibrosis before (MRI1) and after start of lumacaftor/ivacaftor therapy (MRI2-5) in preschool age or school age.** Prevalence n (%) and dominance n (%) of sinus abnormalities are presented on a per-sinus basis, and sinus subscore as mean ± standard deviation.

|  |  |  | MRI1 | MRI2 | MRI3 | MRI4 | MRI5 |
| --- | --- | --- | --- | --- | --- | --- | --- |
| Lumacaftor/ivacaftor from preschool age | **Sphenoid sinus,** n | | 30 | 24 | 22 | 20 | 4 |
|  | **Opacification** | Prevalence, n (%) | 28 (93) | 24 (100) | 22 (100) | 20 (100) | 4 (100) |
|  | **Mucosal swelling** | Prevalence, n (%) | 28 (93) | 24 (100) | 22 (100) | 20 (100) | 4 (100) |
|  |  | Dominance, n (%) | 15 (54) | 17 (71) | 14 (64) | 14 (70) | 4 (100) |
|  | **Mucopyoceles** | Prevalence, n (%) | 15 (50) | 11 (46) | 13 (59) | 7 (35) | 1 (25) |
|  |  | Dominance, n (%) | 13 (46) | 7 (29) | 8 (36) | 6 (30) | 0 (0) |
|  | **Polyps** | Prevalence, n (%) | 0 (0) | 0 (0) | 0 (0) | 0 (0) | 0 (0) |
|  |  | Dominance, n (%) | 0 (0) | 0 (0) | 0 (0) | 0 (0) | 0 (0) |
|  | **Effusion** | Prevalence, n (%) | 0 (0) | 0 (0) | 0 (0) | 0 (0) | 0 (0) |
|  |  | Dominance, n (%) | 0 (0) | 0 (0) | 0 (0) | 0 (0) | 0 (0) |
|  | **Sphenoid sinus subscore** | | **9.3±3.1** | **9.7±1.4** | **9.3±3.2** | **8.9±2.3** | **7.5±1.5** |
| Lumacaftor/ivacaftor from school age | **Sphenoid sinus,** n | | 48 | 42 | 38 | 40 | 18 |
|  | **Opacification** | Prevalence, n (%) | 48 (100) | 39 (93) | 32 (84)** | 34 (85)** | 16 (89) |
|  | **Mucosal swelling** | Prevalence, n (%) | 48 (100) | 39 (93) | 32 (84)** | 34 (85)** | 16 (89) |
|  |  | Dominance, n (%) | 25 (52) | 21 (54) | 20 (63) | 20 (59) | 12 (75) |
|  | **Mucopyoceles** | Prevalence, n (%) | 31 (65) | 25 (60) | 21 (55) | 19 (48) | 8 (44) |
|  |  | Dominance, n (%) | 23 (48) | 18 (46) | 13 (41) | 14 (41) | 4 (25) |
|  | **Polyps** | Prevalence, n (%) | 0 (0) | 0 (0) | 0 (0) | 0 (0) | 0 (0) |
|  |  | Dominance, n (%) | 0 (0) | 0 (0) | 0 (0) | 0 (0) | 0 (0) |
|  | **Effusion** | Prevalence, n (%) | 0 (0) | 0 (0) | 0 (0) | 0 (0) | 0 (0) |
|  |  | Dominance, n (%) | 0 (0) | 0 (0) | 0 (0) | 0 (0) | 0 (0) |
|  | **Sphenoid sinus subscore** | | **10.0±2.1** | **9.0±2.9*** | **8.4±3.5*** | **8.1±3.3*** | **8.0±3.1*** |

**Supplemental Table E5. Chronic rhinosinusitis magnetic resonance imaging (CRS-MRI) scores for the sphenoid sinus in children with cystic fibrosis before (MRI1) and after start of lumacaftor/ivacaftor therapy (MRI2-5) in preschool age or school age.** Prevalence n (%) and dominance n (%) of sinus abnormalities are presented on a per-sinus basis, and sinus subscore as mean ± standard deviation. *p<0.05 vs. MRI1, **p<0.01 vs. MRI1.

|  |  |  | MRI1 | MRI2 | MRI3 | MRI4 | MRI5 |
| --- | --- | --- | --- | --- | --- | --- | --- |
| Lumacaftor/ivacaftor from preschool age | **Ethmoid sinus,** n | | 30 | 24 | 22 | 20 | 4 |
|  | **Opacification** | Prevalence, n (%) | 30 (100) | 23 (96) | 22 (100) | 20 (100) | 4 (100) |
|  | **Mucosal swelling** | Prevalence, n (%) | 30 (100) | 23 (96) | 22 (100) | 20 (100) | 4 (100) |
|  |  | Dominance, n (%) | 17 (57) | 14 (61) | 19 (86)* | 17 (85) | 4 (100) |
|  | **Mucopyoceles** | Prevalence, n (%) | 23 (77) | 19 (79) | 12 (55) | 10 (50) | 2 (50) |
|  |  | Dominance, n (%) | 13 (43) | 9 (39) | 3 (14)* | 3 (15) | 0 (0) |
|  | **Polyps** | Prevalence, n (%) | 0 (0) | 0 (0) | 0 (0) | 0 (0) | 0 (0) |
|  |  | Dominance, n (%) | 0 (0) | 0 (0) | 0 (0) | 0 (0) | 0 (0) |
|  | **Effusion** | Prevalence, n (%) | 0 (0) | 0 (0) | 0 (0) | 0 (0) | 0 (0) |
|  |  | Dominance, n (%) | 0 (0) | 0 (0) | 0 (0) | 0 (0) | 0 (0) |
|  | **Ethmoid sinus subscore** | | **9.4±1.7** | **9.5±2.1** | **8.0±3.1** | **8.7±2.0** | **8.5±0.5** |
| Lumacaftor/ivacaftor from school age | **Ethmoid sinus,** n | | 48 | 42 | 38 | 40 | 18 |
|  | **Opacification** | Prevalence, n (%) | 48 (100) | 42 (100) | 38 (100) | 40 (100) | 18 (100) |
|  | **Mucosal swelling** | Prevalence, n (%) | 48 (100) | 42 (100) | 38 (100) | 40 (100) | 18 (100) |
|  |  | Dominance, n (%) | 32 (67) | 33 (79) | 32 (84) | 32 (80) | 13 (72) |
|  | **Mucopyoceles** | Prevalence, n (%) | 36 (75) | 24 (57) | 21 (55) | 23 (58) | 13 (72) |
|  |  | Dominance, n (%) | 16 (33) | 9 (21) | 6 (16)* | 8 (20)* | 5 (28) |
|  | **Polyps** | Prevalence, n (%) | 0 (0) | 0 (0) | 0 (0) | 0 (0) | 0 (0) |
|  |  | Dominance, n (%) | 0 (0) | 0 (0) | 0 (0) | 0 (0) | 0 (0) |
|  | **Effusion** | Prevalence, n (%) | 0 (0) | 0 (0) | 0 (0) | 0 (0) | 0 (0) |
|  |  | Dominance, n (%) | 0 (0) | 0 (0) | 0 (0) | 0 (0) | 0 (0) |
|  | **Ethmoid sinus subscore** | | **9.3±1.8** | **8.6±2.0** | **8.6±1.8** | **8.9±1.9** | **9.6±1.6** |

**Supplemental Table E6. Chronic rhinosinusitis magnetic resonance imaging (CRS-MRI) scores for the ethmoid sinus in children with cystic fibrosis before (MRI1) and after start of lumacaftor/ivacaftor therapy (MRI2-5) in preschool age or school age.** Prevalence n (%) and dominance n (%) of sinus abnormalities are presented on a per-sinus basis, and sinus subscore as mean ± standard deviation. *p<0.05 vs. MRI1.
